# Healthcare Utilization and Complications Associated With Single-Use Versus Reusable Flexible Cystoscopy in Hospital Outpatient Settings

**DOI:** 10.7759/cureus.91879

**Published:** 2025-09-09

**Authors:** Ben H Chew, Larry E Miller, Kathryn C Morris, Young E Shin, Theodore Tsacogianis, Jenifer White, Sirikan Rojanasarot, Connor M Forbes

**Affiliations:** 1 Department of Urologic Sciences, The University of British Columbia, Vancouver, CAN; 2 Department of Biostatistics, Miller Scientific, Johnson City, USA; 3 Department of Urology, Health Economics and Market Access, Boston Scientific, Marlborough, USA

**Keywords:** cystoscopy, disposable, real-world evidence, reusable, single-use

## Abstract

Purpose

Cystoscopy is one of the most commonly performed urologic procedures in the United States, yet comparative outcome data for single-use versus reusable flexible cystoscopes in hospital outpatient settings are limited. This study compared 30‑day healthcare utilization and complications after single-use versus reusable flexible cystoscopy performed in hospital outpatient settings.

Methods

We performed a retrospective cohort study using the Premier PINC AI Healthcare Database to identify adults who underwent diagnostic cystoscopy in a hospital outpatient setting between January 1, 2022, and August 31, 2024. We compared outcomes of patients who underwent single-use versus reusable flexible cystoscopy. Primary outcomes were 30-day healthcare utilization and complications. Propensity score matching was used to control for demographics, comorbidities, clinical history, and prior healthcare utilization. A predefined subgroup analysis was performed for patients aged 65 years or older.

Results

Of 62,965 eligible encounters, 1,473 (2.3%) used single-use cystoscopes and 61,492 (97.7%) used reusable cystoscopes. After matching (1:5), 1,473 single-use procedures were compared with 7,365 reusable procedures. Thirty-day healthcare utilization was significantly lower with single-use devices (5.2% vs. 13.0%; hazard ratio (HR)=0.39; 95% confidence interval (95% CI): 0.31, 0.49; p<0.001). The results favored single-use cystoscopes for acute care events (p<0.001), emergency department visits (p=0.03), same-day surgeries (p=0.01), and clinic visits (p<0.001). Complication rates were significantly lower with single-use cystoscopes (1.8% vs. 4.3%; HR=0.40; 95% CI: 0.27, 0.60; p<0.001), as were serious complications (HR=0.63, 95% CI: 0.50, 0.81; p<0.001). The findings were similar in patients aged ≥65 years.

Conclusion

In hospital outpatient settings, single-use flexible cystoscopes were associated with substantially lower healthcare utilization and complication rates than reusable devices. These results support considering single-use cystoscopes when planning care pathways in hospital-based outpatient care.

## Introduction

Flexible cystoscopy is one of the most important diagnostic tools in urology. It is fundamental to the investigation of many urologic disorders, such as hematuria, lower urinary tract symptoms, recurrent urinary tract infections (UTIs), and surveillance of urothelial malignancies. In the United States, an estimated three million diagnostic cystoscopies are performed each year [1]. Although complications associated with cystoscopy are generally minor [2], their cumulative burden across this high-volume procedure results in substantial healthcare resource utilization [1,2]. Therefore, optimizing procedural safety while preserving diagnostic performance remains a priority in contemporary urologic practice.

Flexible cystoscopes are classified as semi-critical devices because they contact mucous membranes [3] and are broadly categorized as either reusable or single-use. Reusable cystoscopes are widely used in clinical practice and provide excellent optical quality. However, reusable flexible scopes require ongoing maintenance and meticulous reprocessing between uses to prevent cross-contamination [4], a process that is often inadequately performed [5]. Even with adherence to sterilization guidelines, residual contamination demonstrated by microbial cultures and borescope inspections persists and has raised concerns about infection transmission [6-8]. Repeated use and reprocessing can also degrade these devices by causing surface roughening, stiffening, or channel defects. In a borescope inspection of various endoscopes, scratches were present in 86% of devices, channel shredding in 59%, and intrachannel debris in 23% [9]. Consistent with these findings, Ofstead et al. reported that all 16 examined ureteroscopes had visible irregularities and high contamination rates [10]. Infectious outbreaks have been linked to individual reusable cystoscopes, with the same infectious organisms found in both the device and patient [11].

Single-use cystoscopes are supplied sterile by the manufacturer and discarded after each procedure, which eliminates the risks of inadequate reprocessing and cumulative mechanical degradation. These devices may also provide more consistent deflection and insertion characteristics, providing predictable responsiveness that is important in invasive interventions. Potential drawbacks include higher per-procedure costs and inconsistent findings regarding environmental impact. Although a new device is used for each procedure, this is offset by avoiding the caustic cleaning chemicals such as glutaraldehyde, ortho-phthalaldehyde, and peracetic acid commonly used with reusable scopes [12,13].

Despite the observed risks of reusable cystoscopes, direct comparisons between single-use and reusable flexible cystoscopes using large datasets are lacking. Such a comparison is necessary to adequately detect differences in rare events, such as infectious complications, between the two scope types. Although most cystoscopy procedures in the United States are performed in offices [14], approximately one in four occurs in hospital outpatient settings where higher patient volumes, centralized reprocessing, and multiple operators sharing devices may create operational challenges [1]. In this study, we compared healthcare utilization and complication rates after diagnostic cystoscopy performed with single-use versus reusable flexible cystoscopes in hospital-based outpatient settings using a large national dataset.

## Materials and methods

Ethics

Institutional review board approval and informed consent were not required because the study used de-identified administrative claims data from the Premier PINC AI Healthcare Database (PHD), in accordance with US Title 45 Code of Federal Regulations, Part 46. The PHD complies with the Health Insurance Portability and Accountability Act of 1996 (HIPAA). The study design and reporting adhered to the Strengthening the Reporting of Observational Studies in Epidemiology (STROBE) guidelines for cohort studies [15] and the STROBE extension for propensity score analyses [16].

Data source

The PHD is a United States hospital-based, service-level, all-payer database representing nearly nine million inpatient admissions and more than 86 million outpatient visits annually [17]. The PHD includes standardized billing and clinical fields such as diagnosis and procedure codes, hospital-administered medications, device and supply charges from the charge description master (CDM), and hospital characteristics (e.g., bed size, geographic region, urban/rural location, teaching status). Patients can be tracked longitudinally within a given hospital system using a unique masked identifier.

Study design

This retrospective cohort study evaluated the short-term outcomes of diagnostic flexible cystoscopy performed during hospital outpatient or emergency department (ED) visits. The study period was from January 1, 2021, to September 30, 2024. The patient identification window was from January 1, 2022, to August 31, 2024, where the index date was the first qualifying cystoscopy claim during this period. Patient medical history was assessed during the 365 days prior to the index procedure, and follow-up was ascertained through 30 days post-procedure.

Patients

Eligible patients were aged ≥18 years and underwent diagnostic flexible cystoscopy identified by Current Procedural Terminology (CPT) code 52000 in a hospital-based outpatient clinic (99.7%) or ED (0.3%). Encounters were excluded if additional cystoscopy codes appeared on the same claim or service date, if the procedure occurred in a hospice setting, or if sex or race data were missing. Concurrent diagnoses were identified using International Classification of Diseases, 10th Revision, Clinical Modification (ICD-10-CM) codes.

Exposures

The exposure of interest was the type of cystoscope used in the index procedure. Single-use cystoscopies were identified using universal product numbers (UPNs) or by searching the hospital CDM for product names, company names, and the term “cystoscopy”. Encounters without evidence of a single-use device charge on the index day were classified as reusable cystoscopies.

Outcomes

Primary outcomes were 30-day healthcare utilization and complications. Healthcare utilization included hospitalization, ED visits, same-day surgery, or clinic visits. Complications included sepsis/bacteremia, UTI, hematuria, or urinary retention. We also evaluated two composite endpoints: serious complications (hospitalization, ED visits, same-day surgery, or sepsis/bacteremia) and acute care events (hospitalization, ED visits, same-day surgery, recatheterization, sepsis/bacteremia, UTI, hematuria, or urinary retention).

Data integrity and validation

We applied validated encounter-type codes and diagnostic criteria to all outcome definitions to ensure consistency and minimize misclassification. Analyses were conducted in a secure, de-identified environment using standardized statistical programming. Quality control steps included independent code review, verification of inclusion/exclusion logic, and random sampling of coded events for accuracy.

Propensity score matching

Propensity score matching was used to control for potential confounding factors. Variables in the model were selected based on plausible associations with postoperative outcomes or healthcare utilization. Demographic variables included age, sex, race, and hospital census region. Medical history in the prior year was evaluated for ischemic heart disease, obesity, sepsis, UTI, hematuria, and the Gagne Combined Comorbidity Score [18], while diabetes history was tracked retrospectively from the earliest data available for each patient. Prior healthcare utilization included cystoscopy, number of ED visits, and number of inpatient admissions within one year of the procedure. We used optimal pair matching without replacement, a caliper width of 0.05 on the propensity score logit, and a 1:5 ratio (single‑use:reusable). The probability of undergoing cystoscopy with a single-use device was estimated using binary logistic regression. Model balance was assessed using standardized mean differences (SMDs), with an absolute SMD<0.10 indicating negligible imbalance [19,20].

Statistical analysis

Baseline characteristics and SMDs were reported for the unmatched and matched cohorts. Continuous variables were summarized as means with standard deviations, and categorical variables as counts and percentages. Matched groups were compared using Cox proportional hazards regression, with hazard ratios (HR) and 95% confidence intervals (95% CI) reported. Cumulative incidence plots were generated over the 30-day follow-up period for the primary outcomes. A prespecified subgroup analysis was conducted among propensity score-matched patients aged ≥65 years using the same methods, excluding age from the model. Sensitivity analyses were used to estimate the E-value for the 95% CI to assess the robustness of the primary outcomes. This approach quantifies the minimum strength of association that an unmeasured variable would need to have with both cystoscope type and the outcome to render the findings statistically nonsignificant [21]. Statistical significance was set at a two-sided alpha of 0.05. Analyses were performed using SAS Viya 3.05 (SAS Institute, Cary, NC, USA).

## Results

Patient selection and characteristics

**Figure 1:**
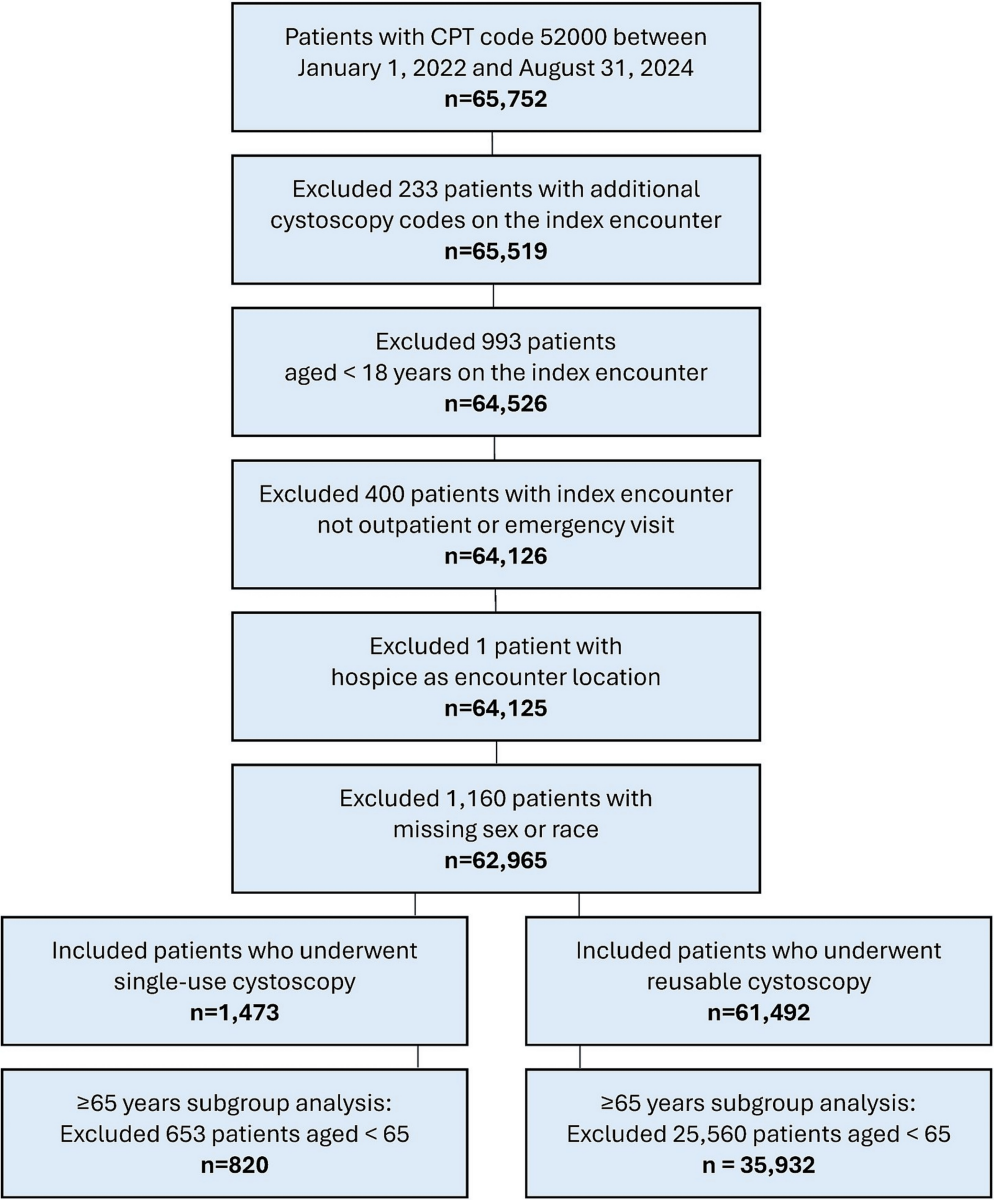
Study cohort selection from the Premier Healthcare Database for cystoscopy procedures performed between January 1, 2022 and August 31, 2024. CPT: Current Procedural Terminology

We identified 65,752 diagnostic cystoscopies performed between January 1, 2022, and August 31, 2024. After exclusions for additional cystoscopy codes on the index encounter, age <18 years, index setting other than outpatient or ED, hospice location, and missing sex or race, 62,965 encounters were included in the main analysis. Of these, 1,473 (2.3%) procedures used a single-use cystoscope, and 61,492 (97.7%) used a reusable cystoscope. The prespecified cohort aged ≥65 years comprised 820 (2.2%) single-use and 35,932 (97.8%) reusable cystoscopes (Figure 1).

After propensity score estimation and 1:5 matching, the main cohort comprised 1,473 single-use procedures matched to 7,365 reusable procedures (Table 1). The ≥65 years subgroup included 820 and 4,100 encounters, respectively (Table 2). In both cohorts, post-matching balance across patient- and hospital-level characteristics was excellent (all absolute SMDs <0.10). In the matched main cohort, mean age was 64±15 vs. 63±15 years (single-use vs. reusable), 59% in each group were male, 84% in each group were White individuals, and most procedures occurred in the Northeastern United States.

**Table 1:** Demographic and clinical characteristics of patients undergoing cystoscopy with single-use or reusable cystoscopes. * Values are expressed as mean±standard deviation or count (percentage). ** Test statistic and p-value obtained from pooled t-test. *** Test statistic and p-value obtained from chi-square test. ED: emergency department; SMD: standardized mean difference; UTI: urinary tract infection

Characteristic*	Unmatched sample					Propensity score matched sample				
	Single-use (N = 1,473)	Reusable (N = 61,492)	Absolute SMD	Test statistic	P-value	Single-use (N = 1,473)	Reusable (N = 7,365)	Absolute SMD	Test statistic	P-value
Age, years**	64 ± 15	64 ± 16	0.05	1.74	0.08	64 ± 15	63 ± 15	0.03	-1.11	0.27
Sex, male***	861 (58.5%)	36,999 (60.2%)	0.03	1.77	0.18	861 (58.5%)	4,367 (59.3%)	0.02	0.36	0.55
Race***										
White individuals	1,235 (83.8%)	46,711 (76.0%)	0.20	49.18	<0.001	1,235 (83.8%)	6,147 (83.5%)	0.00	0.13	0.72
Black individuals	144 (9.8%)	6,350 (10.3%)	0.02	0.47	0.49	144 (9.8%)	727 (9.9%)	0.00	0.01	0.91
Hispanic individuals	61 (4.1%)	5,810 (9.4%)	0.21	47.92	<0.001	61 (4.1%)	332 (4.5%)	0.02	0.39	0.53
Asian individuals	26 (1.8%)	733 (1.2%)	0.05	3.97	0.046	26 (1.8%)	125 (1.7%)	0.01	0.03	0.85
Other	7 (0.5%)	1,888 (3.1%)	0.20	33.19	<0.001	7 (0.5%)	34 (0.5%)	0.00	0.00	0.94
Geographical region***										
Northeast	1,247 (84.7%)	11,014 (17.9%)	1.79	4086.97	<0.001	1,247 (84.7%)	6,195 (84.1%)	0.00	0.27	0.60
South	157 (10.7%)	24,312 (39.5%)	0.71	504.93	<0.001	157 (10.7%)	799 (10.9%)	0.01	0.05	0.83
Midwest	68 (4.6%)	21,390 (34.8%)	0.82	582.80	<0.001	68 (4.6%)	353 (4.8%)	0.00	0.08	0.77
West	1 (0.1%)	4,776 (7.8%)	0.40	121.62	<0.001	1 (0.1%)	18 (0.2%)	0.04	1.78	0.18
Obesity***	52 (3.5%)	6,210 (10.1%)	0.26	69.30	<0.001	52 (3.5%)	205 (2.8%)	0.04	2.42	0.12
Diabetes mellitus***	102 (6.9%)	12,845 (20.9%)	0.41	171.74	<0.001	102 (6.9%)	470 (6.4%)	0.02	0.60	0.44
Ischemic heart disease***	11 (0.7%)	804 (1.3%)	0.06	3.54	0.06	11 (0.7%)	38 (0.5%)	0.03	1.19	0.28
Recent hematuria***	53 (3.6%)	12,749 (20.7%)	0.54	260.74	<0.001	53 (3.6%)	367 (3.6%)	0.00	0.00	0.96
Recent cystoscopy***	35 (2.4%)	5,602 (9.1%)	0.29	80.03	<0.001	35 (2.4%)	134 (1.8%)	0.04	2.03	0.15
Recent sepsis***	14 (1.0%)	1,515 (2.5%)	0.12	13.90	<0.001	14 (1.0%)	56 (0.8%)	0.02	0.56	0.45
Recent UTI***	58 (3.9%)	9,508 (15.5%)	0.40	148.29	<0.001	58 (3.9%)	245 (3.3%)	0.03	1.38	0.24
Comorbidity score**	0.33 ± 1.28	1.02 ± 2.06	0.40	12.81	<0.001	0.33 ± 1.28	0.27 ± 1.09	0.05	-1.93	0.053
No. of ED visits**	0.14 ± 0.53	0.41 ± 1.24	0.28	8.23	<0.001	0.14 ± 0.53	0.12 ± 0.49	0.04	-1.55	0.12
No. of inpatient admissions**	0.10 ± 0.44	0.17 ± 0.58	0.14	4.64	<0.001	0.10 ± 0.44	0.08 ± 0.45	0.04	-1.46	0.14

**Table 2:** Demographic and clinical characteristics of patients aged 65 years and older undergoing cystoscopy with single-use or reusable cystoscopes. * Values are expressed as mean±standard deviation or count (percentage). ** Test statistic and p-value obtained from pooled t-test. *** Test statistic and p-value obtained from chi-square test. ED: emergency department; SMD: standardized mean difference; UTI: urinary tract infection

Characteristic*	Unmatched sample					Propensity score matched sample				
	Single-use (N = 820)	Reusable (N = 35,932)	Absolute SMD	Test statistic	P-value	Single-use (N = 820)	Reusable (N = 4,100)	Absolute SMD	Test statistic	P-value
Age, years**	74 ± 7	75 ± 7	0.07	2.06	0.04	74 ± 7	74 ± 7	0.02	-0.52	0.61
Sex, male***	508 (62.0%)	24,585 (68.4%)	0.14	15.49	<0.001	508 (62.0%)	2,677 (65.3%)	0.07	3.34	0.07
Race***										
White individuals	724 (88.3%)	29,119 (81.0%)	0.20	27.63	<0.001	724 (88.3%)	3,579 (87.3%)	0.00	0.62	0.43
Black individuals	61 (7.4%)	2,895 (8.1%)	0.02	0.41	0.52	61 (7.4%)	342 (8.3%)	0.03	0.74	0.39
Hispanic individuals	21 (2.6%)	2,625 (7.3%)	0.22	27.01	<0.001	21 (2.6%)	123 (3.0%)	0.03	0.46	0.50
Asian individuals	11 (1.3%)	358 (1.0%)	0.03	0.96	0.33	11 (1.3%)	51 (1.2%)	0.01	0.05	0.82
Other	3 (0.4%)	935 (2.6%)	0.19	16.12	<0.001	3 (0.4%)	5 (0.1%)	0.05	2.50	0.11
Geographical region***										
Northeast	685 (83.5%)	5,872 (16.3%)	1.81	2469.48	<0.001	685 (83.5%)	3,413 (83.2%)	0.00	0.04	0.84
South	99 (12.1%)	14,378 (40.0%)	0.67	262.16	<0.001	99 (12.1%)	504 (12.3%)	0.01	0.03	0.86
Midwest	36 (4.4%)	12,777 (35.6%)	0.85	342.97	<0.001	36 (4.4%)	183 (4.5%)	0.00	0.01	0.93
West	0 (0%)	2,905 (8.1%)	0.42	71.98	<0.001	0 (0%)	0 (0%)	0.00	0.00	>0.99
Obesity***	35 (4.3%)	3,552 (9.9%)	0.22	28.72	<0.001	35 (4.3%)	187 (4.6%)	0.01	0.14	0.71
Diabetes mellitus***	83 (10.1%)	9,224 (25.7%)	0.41	102.49	<0.001	83 (10.1%)	428 (10.4%)	0.01	0.07	0.79
Ischemic heart disease***	9 (1.1%)	683 (1.9%)	0.07	2.80	0.09	9 (1.1%)	27 (0.7%)	0.05	1.81	0.18
Recent hematuria***	33 (4.0%)	8,270 (23.0%)	0.58	165.34	<0.001	33 (4.0%)	162 (4.0%)	0.00	0.01	0.92
Recent cystoscopy***	21 (2.6%)	4,202 (11.7%)	0.36	65.76	<0.001	21 (2.6%)	110 (2.7%)	0.01	0.04	0.84
Recent sepsis***	12 (1.5%)	986 (2.7%)	0.09	4.98	0.03	12 (1.5%)	49 (1.2%)	0.02	0.40	0.53
Recent UTI***	39 (4.8%)	6,246 (17.4%)	0.41	90.16	<0.001	39 (4.8%)	150 (3.7%)	0.05	2.23	0.14
Comorbidity score**	0.47 ± 1.50	1.31 ± 2.31	0.43	10.37	<0.001	0.47 ± 1.50	0.45 ± 1.42	0.02	-0.47	0.64
No. ED visits**	0.14 ± 0.52	0.37 ± 1.02	0.29	6.53	<0.001	0.14 ± 0.52	0.15 ± 0.59	0.02	0.61	0.54
No. inpatient admissions**	0.13 ± 0.51	0.20 ± 0.60	0.13	3.39	<0.001	0.14 ± 0.52	0.11 ± 0.48	0.04	-1.02	0.31

The most common ICD-10 diagnosis codes at the time of cystoscopy are listed in Table 3. Distributions were comparable between single-use and reusable groups, with the most common categories being benign prostatic hyperplasia/overactive bladder (BPH/OAB; 40.9% vs. 43.0%), hematuria (31.1% vs. 29.4%), UTI (14.6% vs. 8.7%), malignancy surveillance (12.7% vs. 10.8%), cardiometabolic diseases (11.3% vs. 17.4%), medication-related conditions (10.4% vs. 9.6%), and urologic stone/obstruction (9.2% vs. 9.9%).

**Table 3:** Thirty most common diagnoses at the time of cystoscopy. ICD: International Classification of Diseases

ICD-10 code	ICD description	Percent
N40.1	Benign prostatic hyperplasia with lower urinary tract symptoms	18.1
R31.29	Other microscopic hematuria	15.8
I10	Essential (primary) hypertension	11.8
N40.0	Benign prostatic hyperplasia without lower urinary tract symptoms	9.4
R35.0	Frequency of micturition	9.3
N32.89	Other specified disorders of bladder	9.3
R31.0	Gross hematuria	9.1
Z79.899	Other long-term (current) drug therapy	8.4
Z85.51	Personal history of malignant neoplasm of bladder	7.4
N13.8	Other obstructive and reflux uropathy	7.3
Z87.891	Personal history of nicotine dependence	6.9
E78.5	Hyperlipidemia, unspecified	6.3
Z87.440	Personal history of urinary tract infection	5.9
R31.9	Hematuria, unspecified	5.2
K21.9	Gastro-esophageal reflux disease without esophagitis	5.2
R33.9	Retention of urine, unspecified	5.1
N39.0	Urinary tract infection, site not specified	5.1
Z20.822	Contact with and (suspected) exposure to COVID-19	4.5
N32.81	Overactive bladder	4.5
R39.15	Urgency of urination	4.3
Z08	Encounter for follow-up examination after treatment for malignant neoplasm	4.1
E11.9	Type 2 diabetes mellitus without complications	3.8
R97.20	Elevated prostate-specific antigen (PSA)	3.5
Z79.82	Long-term (current) use of aspirin	3.2
R35.1	Nocturia	3.1
N39.41	Urge incontinence	3.0
F41.9	Anxiety disorder, unspecified	2.8
F17.210	Nicotine dependence, cigarettes, uncomplicated	2.8
E66.9	Obesity, unspecified	2.7
N20.0	Calculus of kidney	2.7

Healthcare utilization

**Figure 2:**
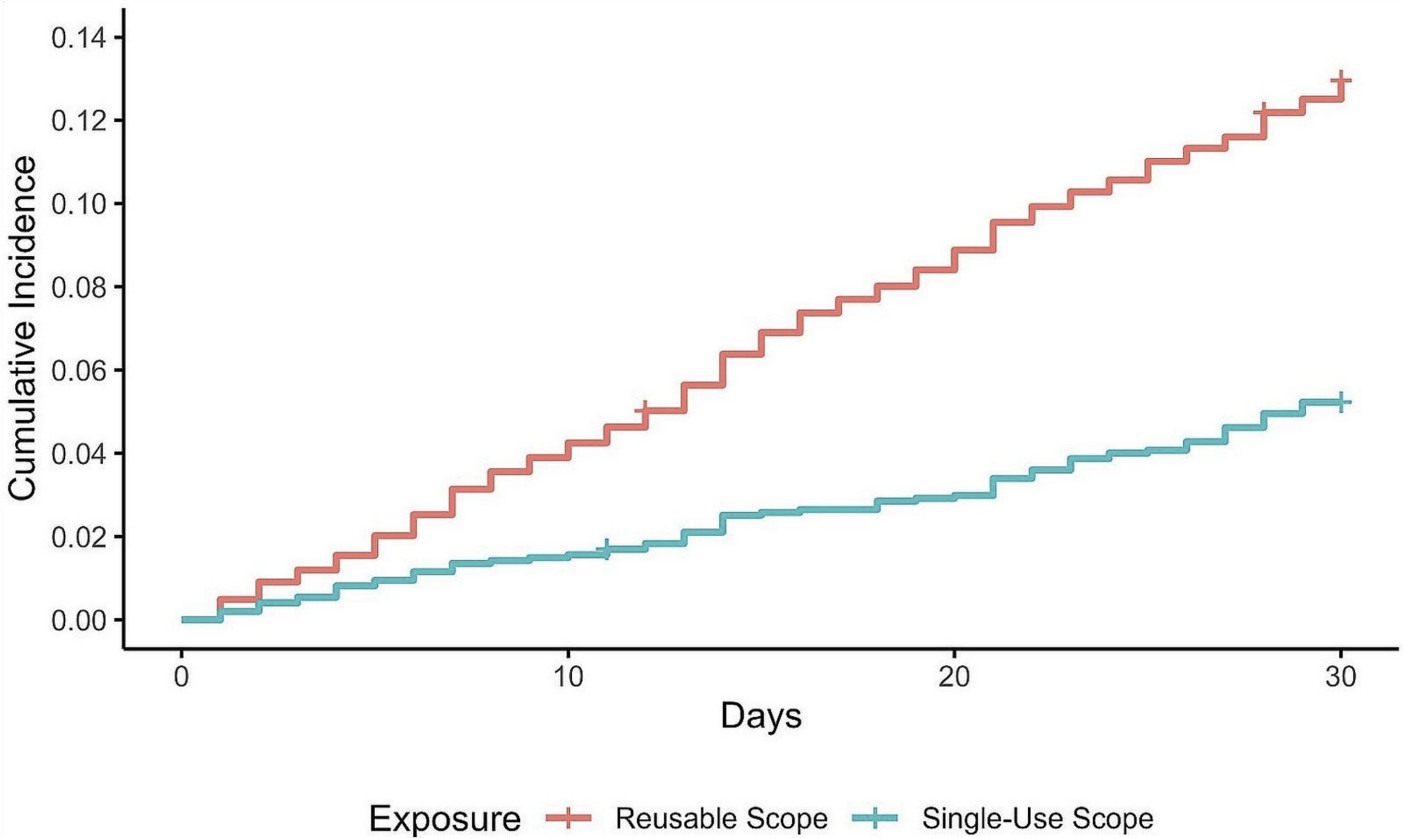
Cumulative 30-day incidence of total healthcare utilization after single-use versus reusable cystoscopy.

Thirty-day healthcare utilization was significantly lower with single-use compared with reusable cystoscopes (HR=0.39; 95% CI: 0.31, 0.49; p<0.001), with a cumulative incidence of 5.2% vs. 13.0%, representing a 61% relative risk reduction (Figure 2). The E-value for the upper bound of the 95% CI was 3.51, indicating that it was very unlikely that the influence of an unmeasured variable could change the primary conclusion. Single-use cystoscopy was associated with a significantly lower relative risk of acute care events (45% reduction; p<0.001), ED visits (40% reduction; p=0.03), same-day surgeries (32% reduction; p=0.01), and clinic visits (97% reduction; p<0.001). Inpatient admissions were numerically lower with single-use cystoscopy (43% reduction) but not statistically different between groups (p=0.07).

**Figure 3:**
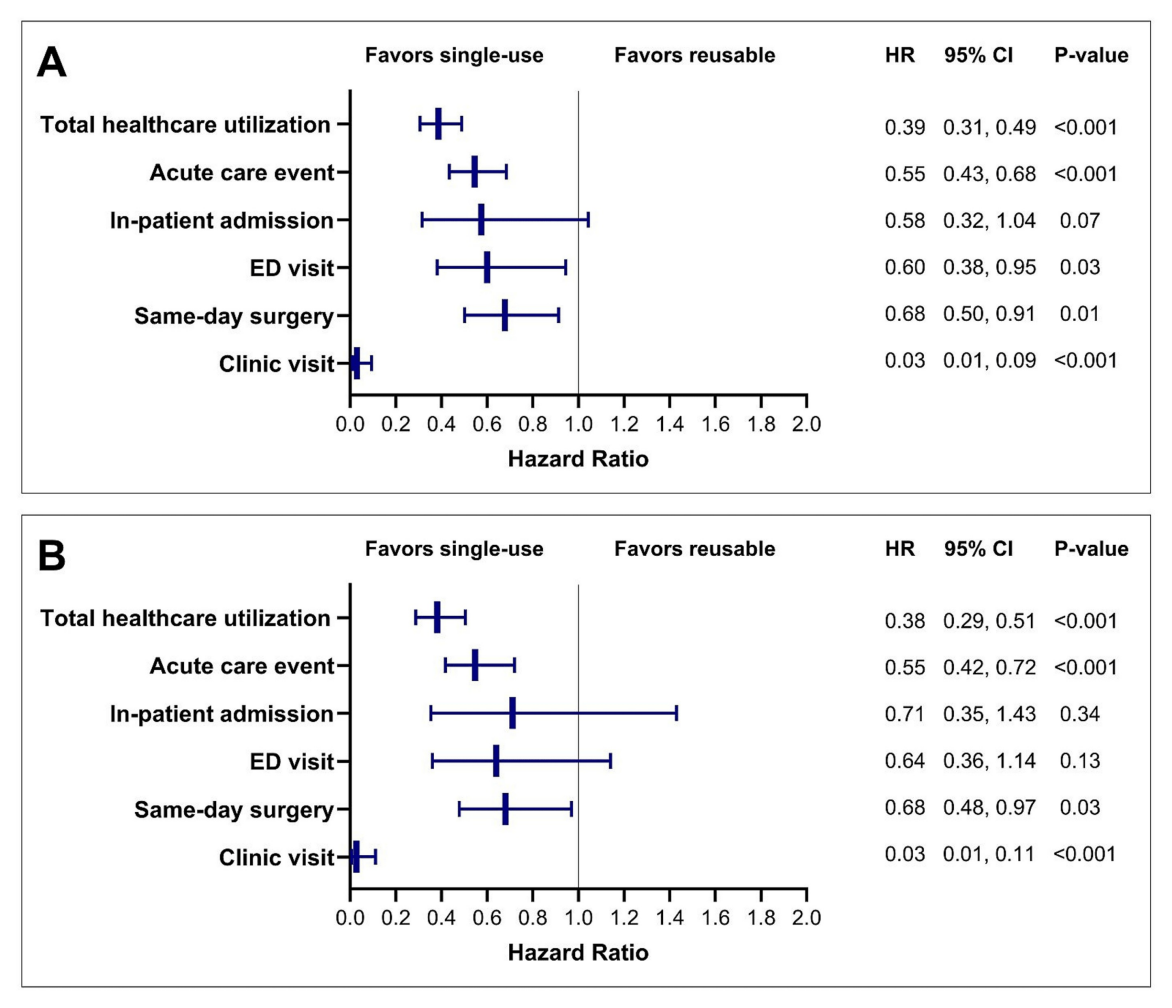
Healthcare utilization over 30 days after single-use versus reusable cystoscopy in the overall cohort (A) and the ≥65 years subgroup (B). The plot depicts hazard ratios (HR) and 95% confidence intervals (95% CI). An HR <1.0 indicates lower risk with single-use cystoscopy compared to reusable cystoscopy.

The findings were similar in the ≥65 years subgroup, with a 30-day healthcare utilization cumulative incidence of 6.3% vs. 15.7%, representing a 62% relative risk reduction with single-use cystoscopes (HR=0.38; 95% CI: 0.29, 0.51; p<0.001). Single-use cystoscopy was associated with a significantly lower relative risk of acute care events (45% reduction; p<0.001), same-day surgeries (32% reduction; p=0.03), and clinic visits (97% reduction; p<0.001), with no statistical differences in inpatient admissions (29% risk reduction; p=0.34) or ED visits (36% risk reduction; p=0.13) (Figure 3).

Complications

**Figure 4:**
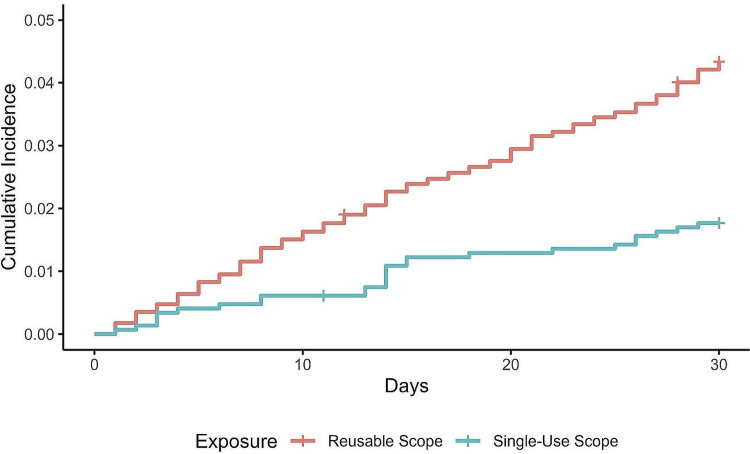
Cumulative 30-day incidence of all complications after single-use versus reusable cystoscopy.

Overall, 30-day complication rates were significantly lower with single-use devices. The cumulative incidence was 1.8% vs. 4.3% (Figure 4), representing a 60% relative risk reduction (HR=0.40; 95% CI: 0.27, 0.60; p<0.001). The E-value for the upper bound of the 95% CI was 2.71, indicating that it was very unlikely that the influence of an unmeasured variable could change the primary conclusion. Serious complication rates also favored single-use devices (HR=0.63; 95% CI: 0.50, 0.81; p<0.001), corresponding to a 37% risk reduction. Single-use devices were associated with a lower relative risk of urinary retention (65% reduction; p<0.001) and hematuria (66% reduction; p=0.003), with no statistical differences in recatheterization (47% risk reduction; p=0.11), UTI (6% risk increase; p=0.85), or sepsis/bacteremia (87% risk reduction; p=0.17).

**Figure 5:**
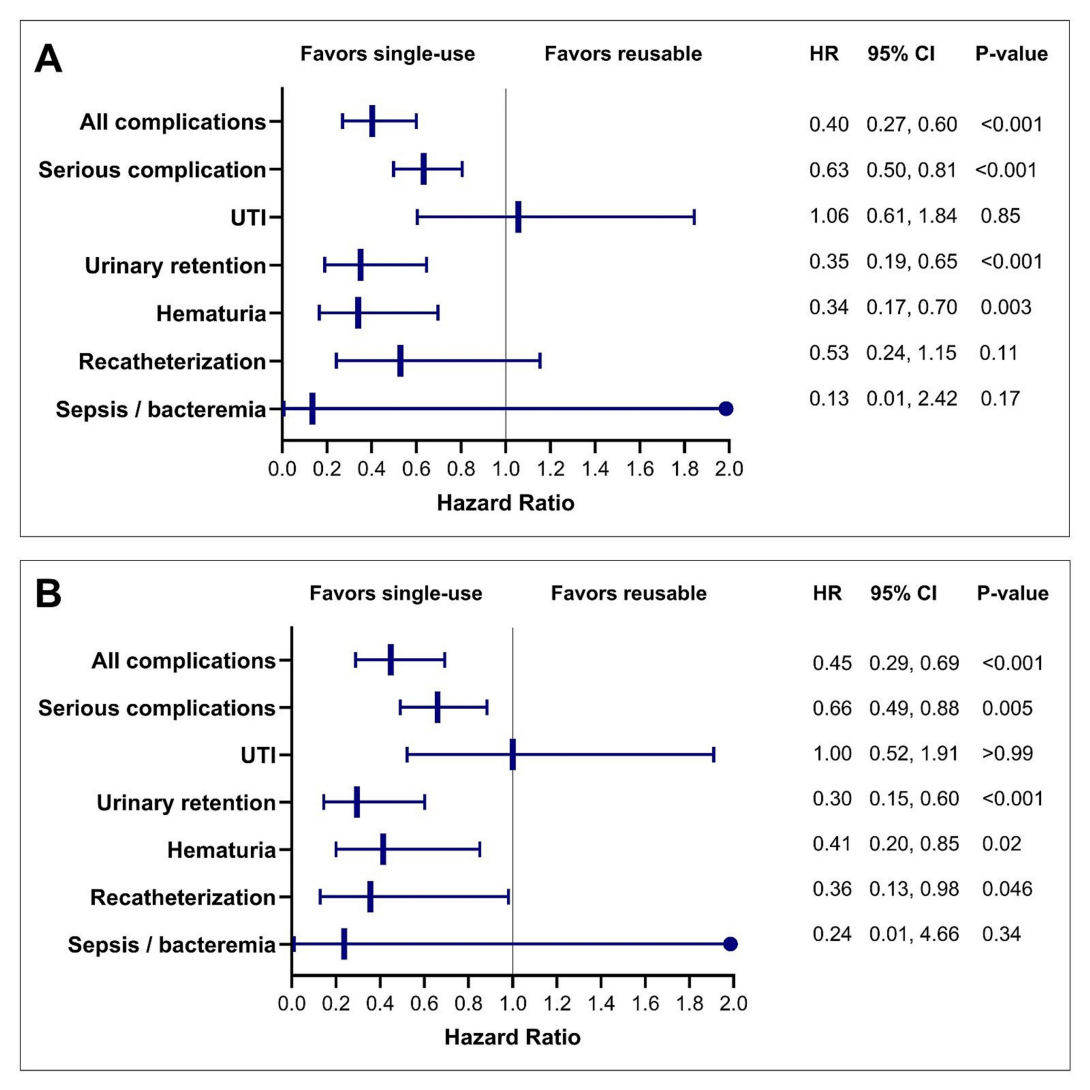
Complications over 30 days after single-use versus reusable cystoscopy in the overall cohort (A) and the ≥65 years subgroup (B). The plot depicts hazard ratios (HR) and 95% confidence intervals (95% CI). An HR <1.0 indicates lower risk with single-use cystoscopy compared to reusable cystoscopy. UTI: urinary tract infection

In the ≥65 years subgroup, the results paralleled the main cohort. The relative risks of complications (2.7% vs. 5.9%; 55% risk reduction; p<0.001), serious complications (34% risk reduction; p=0.005), urinary retention (70% risk reduction; p<0.001), hematuria (59% risk reduction; p=0.02), and recatheterization (64% risk reduction; p=0.046) were significantly lower with single-use devices. The risks of UTI (0% risk reduction; p>0.99) and sepsis/bacteremia (76% risk reduction; p=0.34) did not differ between the groups (Figure 5).

## Discussion

Among adults undergoing diagnostic cystoscopy in hospital outpatient settings, single-use flexible cystoscopes were associated with a 61% relative reduction in healthcare utilization and a 60% relative reduction in complications compared with reusable devices. Single-use devices were also associated with lower risks of serious complications, acute care events, ED visits, same-day surgeries, and clinic visits, with no differences in infectious complications such as UTI and sepsis/bacteremia. The results were consistent in patients aged ≥65 years, suggesting that the benefits extend across age groups. Collectively, these findings support considering single-use flexible cystoscopes when developing care pathways for hospital outpatient settings.

The comparable infection rates between device types challenge assumptions that cystoscope-related complications are primarily infectious. Prior work has emphasized contamination and infection risks with reusable instruments, but the findings have been mixed. A systematic review of 21 studies reported no differences in overall complications or infection rates between single-use and reusable urologic endoscopes [22]; however, the small sample sizes (median 131 patients) limited the power to detect differences. In contrast, in a propensity score-matched sample of nearly 1,000 office-based procedures, Geldmaker et al. reported a lower risk of 30-day positive urine cultures and fewer unplanned encounters with single-use versus reusable cystoscopy [23]. Furthermore, Unno et al. reported that while overall complication rates were comparable with single-use and reusable ureteroscopes, the odds of postoperative UTI were 63% lower with single-use devices [24]. In our matched cohort of over 8,800 patients treated in hospital outpatient settings, overall complication rates were lower with single-use devices, yet infectious complications (e.g., UTI, sepsis, bacteremia) did not differ between groups. Overall, these findings suggest that noninfectious factors may contribute to postprocedural morbidity, although an infectious component cannot be ruled out and may be less likely with single-use devices in some settings. Subclinical infectious processes may also contribute to symptoms such as hematuria without producing a positive urine culture required for infection diagnosis. Prospective studies should explore the associations among device contamination levels and surface characteristics, insertion forces, mucosal changes, and clinical outcomes. These studies should also aim to measure and adjust for potential confounders such as insurance type or household income, which may affect healthcare access and follow-up and contribute to differences in clinical outcomes.

Our results are concordant with FDA communications and professional society guidance highlighting concerns about flexible cystoscope reprocessing [6,7], and with an analysis of over 90,000 endoscope samples in which 13% of devices in use should have been quarantined for excess contamination [25]. These advisories not only emphasized the risks of contamination and the need for strict adherence to reprocessing protocols but also warned against using devices with damaged channels, kinks, distal-end imperfections, or other wear. Even with proper processing, physical degradation may increase this risk [9]. Reprocessing itself contributes to device damage, with flexible cystoscopes requiring repair after approximately every 15 uses at a mean cost exceeding $6,800 per repair [4]. Notably, nearly three out of four urologic endoscope repairs result from damage incurred during reprocessing rather than clinical use [26]. This cycle of damage and repair likely contributes to the surface irregularities and associated mechanical changes reported in more than 85% of devices [9,10]. Together with our finding that over 75% of patients with a complication had a downstream utilization encounter, this suggests that short-term healthcare use after cystoscopy may be linked to device characteristics. For hospital systems that routinely perform cystoscopy, fewer return visits with single-use devices may improve throughput, whereas device costs may be offset by eliminating reprocessing and repair expenses [27-29].

Strengths of this study include a large, heterogeneous national sample, propensity score matching to address confounding, and a prespecified ≥65-year subgroup showing results consistent with the main cohort. This study also had several limitations that warrant mention. First, the device type was identified from hospital charge records and could be misclassified if charges were bundled or missing. Additionally, it is plausible that complication encounters in both groups could have been miscoded as the primary presenting symptom, such as hematuria, when the underlying cause was actually infectious. Second, patient factors (e.g., insurance type, household income, urine culture testing), procedural details (e.g., practice patterns, operator experience), and device details (e.g., sterilization methods for reusable cystoscopes) that may have influenced outcomes were not evaluated in this study. However, it is unlikely that these factors would have altered the overall study conclusions given the sensitivity analysis results. Third, analyses were restricted to hospital-based outpatient settings. Although most (70%) diagnostic cystoscopies in the United States are performed in office settings [14], these encounters generally involve lower-risk patients with low expected health utilization and complication rates. In contrast, the settings evaluated in this study account for approximately 25% of national procedures [1] and involve higher-risk patients with more concurrent comorbidities. Interestingly, in the unmatched sample, only 2.3% of procedures used single-use cystoscopes, which reflects their limited adoption during the study period despite reports that 88-95% of patients expressed a preference for single-use devices [30,31]. Whether the benefits observed with single-use devices in this study would translate to office-based procedures remains unclear; however, some evidence suggests that these advantages may apply to office cystoscopy [23]. Finally, follow-up was limited to 30 days post-procedure. While this restriction was implemented because events beyond this period are less likely to be related to cystoscopy, late events may have occurred and were thus excluded from our analysis.

## Conclusions

In hospital outpatient settings, single-use flexible cystoscopes were associated with a 61% relative reduction in healthcare utilization and a 60% relative reduction in complications compared with reusable devices. The results in patients aged ≥65 years were comparable to those of the main cohort, suggesting that the benefits extend across age groups. These results support considering single-use flexible cystoscopes when planning care pathways in hospital-based outpatient care. Their use may reduce unplanned follow-up visits, eliminate reprocessing requirements, and avoid repair costs associated with reusable devices. The main strength of this study was the use of a propensity-score matched national sample, while the main limitation was the potential risk of residual confounding. Future prospective studies are needed to confirm these findings, assess longer-term outcomes, explore the applicability of single-use devices to other procedural settings such as office-based cystoscopy, and determine whether the observed differences are related to device characteristics or procedural factors.

## References

[1] (2025). Atlas dataset. https://www.definitivehc.com/why-definitive-healthcare/our-platform/atlas-dataset.

[2] Stav K, Leibovici D, Goren E, Livshitz A, Siegel YI, Lindner A, Zisman A (2004). Adverse effects of cystoscopy and its impact on patients' quality of life and sexual performance. Isr Med Assoc J.

[3] Spaulding EH (1968). Chemical disinfection of medical and surgical materials. Disinfection, Sterilization and Preservation.

[4] Rindorf DK, Tailly T, Kamphuis GM, Larsen S, Somani BK, Traxer O, Koo K (2022). Repair rate and associated costs of reusable flexible ureteroscopes: a systematic review and meta-analysis. Eur Urol Open Sci.

[5] Redin MR, Perez CD, Valdes CB, Jose-Saras DS, Moreno-Nunez P, Vicente-Guijarro J, Aranaz-Andres JM (2025). Ensuring patient safety during cystocopy: risk assessment of device reprocessing through healthcare failure mode and effects analysis. World J Urol.

[6] Clemens JQ, Dowling R, Foley F (2010). Joint AUA/SUNA white paper on reprocessing of flexible cystoscopes. J Urol.

[7] Jaklevic MC (2021). Reprocessed urological endoscopes tied to infection risk. JAMA.

[8] Ofstead CL, Smart AG, Hurst LL, Lamb LA (2025). Endoscope processing effectiveness: a reality check and call to action for infection preventionists and clinicians. Am J Infect Control.

[9] Thaker AM, Kim S, Sedarat A, Watson RR, Muthusamy VR (2018). Inspection of endoscope instrument channels after reprocessing using a prototype borescope. Gastrointest Endosc.

[10] Ofstead CL, Heymann OL, Quick MR, Johnson EA, Eiland JE, Wetzler HP (2017). The effectiveness of sterilization for flexible ureteroscopes: a real-world study. Am J Infect Control.

[11] Jeffrey L, Elie KM, Dhaval J, Daniel T, Ariel S (2022). Post-cystoscopy infections and device malfunctions in reprocessed flexible cystoscopes in a national database. Can J Urol.

[12] Kemble JP, Winoker JS, Patel SH, Su ZT, Matlaga BR, Potretzke AM, Koo K (2023). Environmental impact of single-use and reusable flexible cystoscopes. BJU Int.

[13] Jahrreiss V, Sarrot P, Davis NF, Somani B (2024). Environmental impact of flexible cystoscopy: a comparative analysis between carbon footprint of Isiris(R) single-use cystoscope and reusable flexible cystoscope and a systematic review of literature. J Endourol.

[14] (2025). CMS-1832-P. https://www.cms.gov/medicare/payment/fee-schedules/physician/federal-regulation-notices/cms-1832-p.

[15] von Elm E, Altman DG, Egger M (2007). The Strengthening the Reporting of Observational Studies in Epidemiology (STROBE) statement: guidelines for reporting observational studies. Lancet.

[16] Yao XI, Wang X, Speicher PJ (2017). Reporting and guidelines in propensity score analysis: a systematic review of cancer and cancer surgical studies. J Natl Cancer Inst.

[17] (2025). Premier healthcare database: data that informs and performs (white paper). https://offers.premierinc.com/rs/381-NBB-525/images/PremierHealthcareDatabaseWhitepaper.pdf.

[18] Gagne JJ, Glynn RJ, Avorn J, Levin R, Schneeweiss S (2011). A combined comorbidity score predicted mortality in elderly patients better than existing scores. J Clin Epidemiol.

[19] Hripcsak G, Zhang L, Chen Y, Li K, Suchard MA, Ryan PB, Schuemie MJ (2025). Assessing covariate balance with small sample sizes. Stat Med.

[20] Austin PC (2009). Balance diagnostics for comparing the distribution of baseline covariates between treatment groups in propensity-score matched samples. Stat Med.

[21] VanderWeele TJ, Ding P (2017). Sensitivity analysis in observational research: introducing the E-value. Ann Intern Med.

[22] Anderson S, Patterson K, Skolarikos A, Somani B, Bolton DM, Davis NF (2024). Perspectives on technology: to use or to reuse, that is the endoscopic question-a systematic review of single-use endoscopes. BJU Int.

[23] Geldmaker LE, Baird BA, Lyon TD (2023). Conversion to disposable cystoscopes decreased post-procedure encounters and infections compared to reusable cystoscopes. Urol Pract.

[24] Unno R, Hosier G, Hamouche F, Bayne DB, Stoller ML, Chi T (2023). Single-use ureteroscopes are associated with decreased risk of urinary tract infection after ureteroscopy for urolithiasis compared to reusable ureteroscopes. J Endourol.

[25] Pineau L (2023). Endoscope reprocessing: retrospective analysis of 90,311 samples. Endosc Int Open.

[26] Sooriakumaran P, Kaba R, Andrews HO, Buchholz NP (2005). Evaluation of the mechanisms of damage to flexible ureteroscopes and suggestions for ureteroscope preservation. Asian J Androl.

[27] Kim J, Gao B, Bhojani N, Zorn KC, Chughtai B, Elterman DS (2022). Micro-cost analysis of single-use vs. reusable cystoscopy in a single-payer healthcare system. Can Urol Assoc J.

[28] Su ZT, Huang MM, Matlaga BR, Hutfless S, Koo K (2021). A micro-costing analysis of outpatient flexible cystoscopy: implications for adoption of single-use flexible cystoscopes. World J Urol.

[29] Kenigsberg AP, Gold S, Grant L, Lotan Y (2021). The economics of cystoscopy: a microcost analysis. Urology.

[30] Borja Brugés CN, Rindorf DK, Cepeda M, Schultz Hansen K (2022). Willingness to pay and preferences among patients undergoing cystoscopies: results from a large survey-based study in Spain. Res Rep Urol.

[31] Wong A, Phan Y, Thursby H, Mahmalji W (2021). The first UK experience with single-use disposable flexible cystoscopes: an in-depth cost analysis, service delivery and patient satisfaction rate with Ambu® ascope™ 4 cysto. JELEU.

